# Was It Dilatation of Left Ventricle?

**Published:** 1880-04

**Authors:** K. J. Thomas

**Affiliations:** Greenwood, Wis.


### ARTICLE IX.

*Was it Dilatation of Left Ventricle?*

MESSRS. EDITORS:—I ask the above question and would like through your columns to get some ideas from some of your readers who take more special interest in heart troubles than I have done. February 22d, I was called to see John H——, previously as hearty, strong and robust a man as could be found in the State. His statement was, that two days before, while chopping down pine, he began racing with another party and worked all day exerting himself to his utmost. That night he did not sleep at all but had no pain or distress; next day he went to work but was obliged to stop at noon, and went home. It was the following morning that I saw him. He presented the appearance of a person with general dropsy in the last stages of valvular disease of the heart. His legs, feet, hands, arms, and even face were as distended as they could well be; and all this effusion came on in one night only. He complained of some distress for breath. Respiration was slow, and pulse 30. I examined the heart and found but a slight murmur, or whistling sound with the second sound of the heart. The heart's action was labored but regular, the left ventricle taking a long time to contract. Pulse although only 30 per minute, was very full and strong. I could count it by watching the tremor or jar of the whole arm. I put him on syr. iodide of iron; also gave iodide of potassium. For diuretics used pot. nitrate and pot. acet. on alternate days; used calomel and jalap as hydragogue cathartic each night. Under this treatment the third day the pulse was 45; sixth day 58, gradually improving until to-day pulse is 70, with but slight anasarca towards evening. I have examined no less than twelve different authors on cardiac diseases and cannot thus far find a similar case, and in fourteen years practice I have never seen anything resembling it. I had good counsel in the case, and it was also new to the gentlemen summoned.

K. J. THOMAS, M.D.

GREENWOOD, WIS., March 12, 1880.

---

THE editor of the *Journal of Nervous and Mental Diseases* will pay seventy-five cents each for copies of the first issue of that publication—January, 1874. Address, Dr. J. S. Jewell, 70 Monroe Street.

